# High-energy particle observations from the Moon

**DOI:** 10.1098/rsta.2023.0311

**Published:** 2024-05-09

**Authors:** Iannis Dandouras, Elias Roussos

**Affiliations:** ^1^ Institut de Recherche en Astrophysique et Planétologie, Université de Toulouse/CNRS/UPS/CNES, Toulouse, France; ^2^ Max Planck Institute for Solar System Research, Goettingen, Germany

**Keywords:** Moon, deep space, space plasmas, high-energy particles, heliophysics, space weather

## Abstract

The Moon is a unique natural laboratory for the study of the deep space plasma and energetic particles environment. During more than 3/4 of its orbit around the Earth it is exposed to the solar wind. Being an unmagnetized body and lacking a substantial atmosphere, solar wind and solar energetic particles bombard the Moon's surface, interacting with the lunar regolith and the tenuous lunar exosphere. Energetic particles arriving at the Moon's surface can be absorbed, or scattered, or can remove another particle from the lunar regolith by sputtering or desorption. A similar phenomenon occurs also with the galactic cosmic rays, which have fluxes and energy spectra representative of interplanetary space. During the remaining part of its orbit the Moon crosses the tail of the terrestrial magnetosphere. It then provides the opportunity to study *in-situ* the terrestrial magnetotail plasma environment as well as atmospheric escape from the Earth's ionosphere, in the form of heavy ions accelerated and streaming downtail. The lunar environment is thus a unique natural laboratory for analysing the interaction of the solar wind, the cosmic rays and the Earth's magnetosphere with the surface, the immediate subsurface, and the surface-bounded exosphere of an unmagnetized planetary body.

This article is part of a discussion meeting issue ‘Astronomy from the Moon: the next decades (part 2)’.

## 1. Introduction

The Moon is a unique natural laboratory for the study of the deep space plasma and energetic particles environment. During more than 3/4 of its orbit around the Earth the Moon is directly exposed to the solar wind. Lacking a global intrinsic magnetic field and without a collisional atmosphere, solar wind and solar energetic particles (SEPs) arrive nearly without any deviation or absorption and bombard the Moon's surface, interacting with the lunar regolith and the tenuous lunar exosphere [1–5]. A similar phenomenon occurs also with the galactic cosmic rays (GCRs), which have fluxes and energy spectra representative of interplanetary space [6]. Downstream from the Moon a wake is formed consisting of a structured plasma umbra and penumbra area, showing a gradual decrease of the plasma density [7,8].

The Moon's environment is thus an ideal location to study galactic cosmic rays, solar wind and solar energetic particles. This environment is typical of deep space [9], aside from the fact that the Moon itself presents an obstacle to the GCRs and also interacts with them.

**Figure 1.**
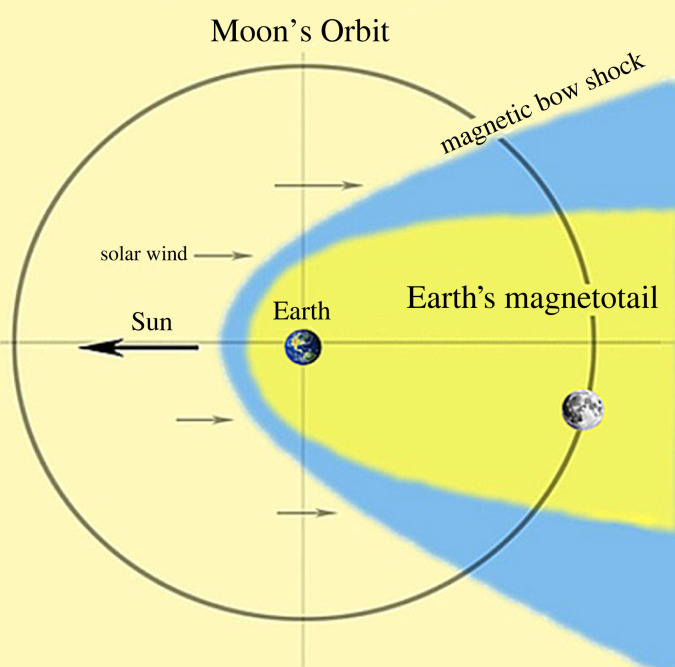
Moon's orbit with respect to the Earth's magnetosphere. Earth's and Moon's sizes are not to scale. (Adapted from: Tim Stubbs/University of Maryland/GSFC).

During the remaining part of its orbit the Moon crosses the tail of the terrestrial magnetosphere (figure 1). During these periods it is not exposed to the solar wind but to the terrestrial magnetotail plasma environment, providing the opportunity to study *in-situ*, from the Moon or from an observational platform in lunar orbit, the dynamics of the magnetotail and its dependence on drivers such as the solar and geomagnetic activity conditions [10]. Phenomena such as for instance plasmoids released from the near-Earth magnetotail and propagating anti-Sunward, hot plasma flows, energetic particle bursts, plasma waves, magnetic reconnection and plasma sheet dynamics can then be observed *in-situ* [11–16].

The Moon during these periods is also very well placed to monitor atmospheric escape from the Earth into space [17–21], in the form of energetic heavy ions outflowing from the terrestrial ionosphere and transported and lost into the deep magnetotail. The observations provided by the THEMIS-ARTEMIS and from the Kaguya (SELENE) spacecraft confirmed the presence of such ions, of terrestrial origin, in the environment of the Moon [22,23].

**Figure 2.**
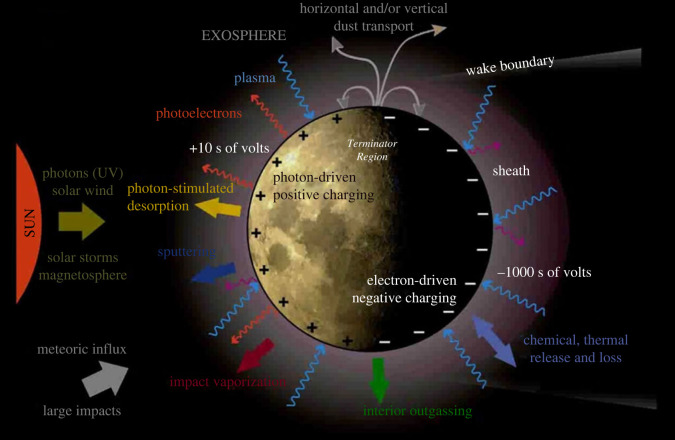
Moon's environment with the complex interaction between solar radiation, space plasma, meteoritic flux, dust, exosphere and the surface (Credit: Jasper Halekas).

The lunar environment offers the opportunity to use our closest neighbour in order to study the surface-bounded exosphere of an ‘airless’ unmagnetized planetary body (figure 2), its production mechanisms, its dynamics, its interaction with the solar wind and with the Earth's magnetosphere plasma, and its escape into space [4,24–27]. The sources of the lunar exosphere include the solar wind, the release of atoms from the regolith through diverse interaction mechanisms (thermal release, photon stimulated desorption, electron stimulated desorption, sputtering, micrometeorite impact vaporization, etc.), and lunar outgassing [24,25,28]. The LADEE (Lunar Atmosphere and Dust Environment Explorer) and LRO (Lunar Reconnaissance Orbiter) observations have shown the complexity of the lunar exosphere and of the associated physical processes [29–31].

The lunar surface is also subject to energetic ion implantation into the regolith [32–36]. The interaction of SEPs and GCRs with the Moon's regolith can produce albedo energetic particles [37–39], whereas the interaction of the solar wind with the regolith can give rise to ion implantation or neutralization and reflection from the regolith surface [40–43]. The same interaction can also lead to the formation of hydrogen bearing molecules [29,44,45], possibly including hydroxyl radicals and eventually water [46,47]. Solar wind interaction with crustal magnetic anomalies [48–50], lunar pickup ion generation [51,52], or lunar surface electrostatic charging and dust levitation [53,54], are just some more paradigms of the physical processes taking place in the plasma environment of the Moon.

The analysis of particles implanted in the lunar regolith, which originated from the Earth's atmosphere, can also provide some information on the early terrestrial atmosphere [26,32,55,56]. Planetary evolution models suggest that the early terrestrial atmosphere experienced an intense escape of hydrogen, oxygen and carbon, resulting from the dissociation of water and methane molecules, and of nitrogen due to the increased EUV flux from the active young Sun [55,57–60]. As suggested by Marty *et al*. [56], nitrogen originating from the early Earth has been implanted and preserved in the lunar regolith.

In addition, the Moon's regolith represents the archetype of a planetary surface subject to space weathering, which results from its exposure to energetic particles, i.e. surface–energetic particle interactions [61–64]. As the irradiation of the Moon by GCRs is almost uniform, any variation in the emitted albedo particles is expected to be the result of the physical or chemical phenomena occurring at its surface or immediate sub-surface [37,65].

Succeeding to the legendary Apollo missions of the late 1960s–early 1970s, to the Luna missions, and to the more recent missions to the Moon (THEMIS-ARTEMIS, Kaguya, LADEE, LRO, Chang'E, Chandrayaan, etc.), a ‘new wave’ of lunar missions is under preparation, drawing from their legacy [66–68]. Of particular interest is the Lunar Orbital Platform - Gateway (or just Gateway), a manned space station prepared under international collaboration and which will be assembled into a halo orbit around the Moon. The Gateway will offer unprecedented scientific payload capacity in lunar orbit.

## 2. Energetic particle environment of the Moon

While there is no generally accepted definition of what can classify a particle as ‘energetic’, in the context of the Moon–charged particle interactions we refer to those particles that typically have the potential to drive changes in the physical and/or chemical state of the lunar exosphere, or of the lunar surface, or of the immediate subsurface. This definition would then include solar wind ions starting from the low-keV energy range and extending up to energies as high as those of the Galactic Cosmic Rays (GCRs).

### (a) Moon in the solar wind

As the Moon has neither a global intrinsic magnetic field nor a collisional atmosphere, its surface is exposed to:

— Solar Wind: approximately 0.5 to 10 keV ions (and lower energy electrons)
— Solar Energetic Particles (SEPs): approximately 10 keV to several 100 MeV ions and electrons
— Galactic Cosmic Rays (GCRs): approximately 100 MeV to approximately 10 GeV ions and electrons
— Anomalous Cosmic Rays (ACRs): approximately 1 to approximately 100 MeV particles

The separation in energy is only approximate and indicates the order of magnitude of the energy range for each population.

Monitoring the solar wind (e.g. [69]) in the lunar environment allows us to assess its impact on the dynamics of the Earth's and the Moon's exospheres, on the dynamics of the Earth's magnetosphere, and on the sputtering, electrostatic charging and weathering of the Moon's surface. This can become of paramount importance during severe space weather events, when the solar wind and SEP parameters can take extreme values [70–73].

Monitoring and characterizing the SEPs and GCRs allows us to assess the radiation environment of the Moon, in view of the upcoming return of humans to the Moon and of the related radiation risks. It also allows us to assess the role of SEPs and GCRs as lunar surface sputtering sources and for the production of cosmogenic nuclides on the subsurface, the concentration of which is critical for dating samples. Since the Moon does not have a global magnetic field it is possible, with an appropriate particle detector, to measure the low-energy component of the GCR spectrum (less than 1 GeV) with high precision. This provides an advantage with respect to low-Earth orbits, where most of the advanced GCR observatories like PAMELA and AMS-02 are situated, where this low energy part is (partially) filtered out by the Earth's magnetosphere and it can also get mixed with trapped radiation belt species, or with GCR albedo particles from the Earth's atmosphere.

**Figure 3.**
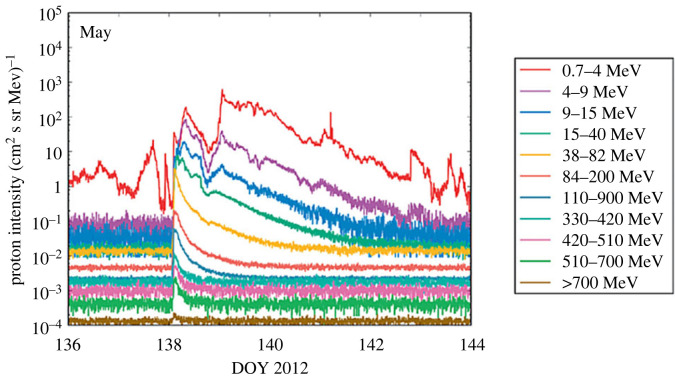
Typical SEP (Solar Energetic Particles) proton intensities: five-minute averages of proton intensities measured at the geostationary orbit by GOES-13/EPS/HEPAD during the May 2012 solar events. (From: [74]).

Typical SEP proton fluxes, measured during a solar event, are shown in figure 3 (adapted from [74]). Some of the SEP protons (∼MeV energy range) can also interact in the high solar corona with partially stripped coronal ions, charge exchange with them, and produce ∼MeV ENAs (Energetic Neutral Atoms) [75].

**Figure 4.**
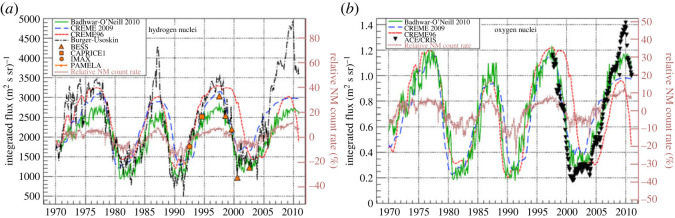
Typical GCR (Galactic Cosmic Rays) hydrogen nuclei (*a*) and oxygen nuclei (*b*) fluxes. (Adapted from: [76]).

GCR hydrogen and oxygen nuclei fluxes are shown in figure 4, presenting a clear solar cycle modulation (adapted from [76]).

Anomalous Cosmic Rays (ACRs) are pickup ions derived from interstellar medium atoms, which have been ionized through either charge exchange interactions with the solar wind or photo-ionization by the solar UV radiation. Then they have been successively accelerated at the termination shock and transported inwards in the heliosphere. They appear in the GCR spectrum as an ‘anomalous’ increase at the lower energies, of the order of approximately MeV – approximately 100 MeV [77].

### (b) Moon in the terrestrial magnetosphere

When the Moon crosses the tail of the Earth's magnetosphere, it is exposed not to the solar wind but to the terrestrial plasma sheet and to the plasma sheet boundary layer. The Moon's plasma environment is then dominated by the Earth's magnetotail magnetic field and energetic ions and electrons, including energetic ions originating from the terrestrial ionosphere, accelerated in the near-Earth magnetosphere and then streaming downtail.

**Figure 5.**
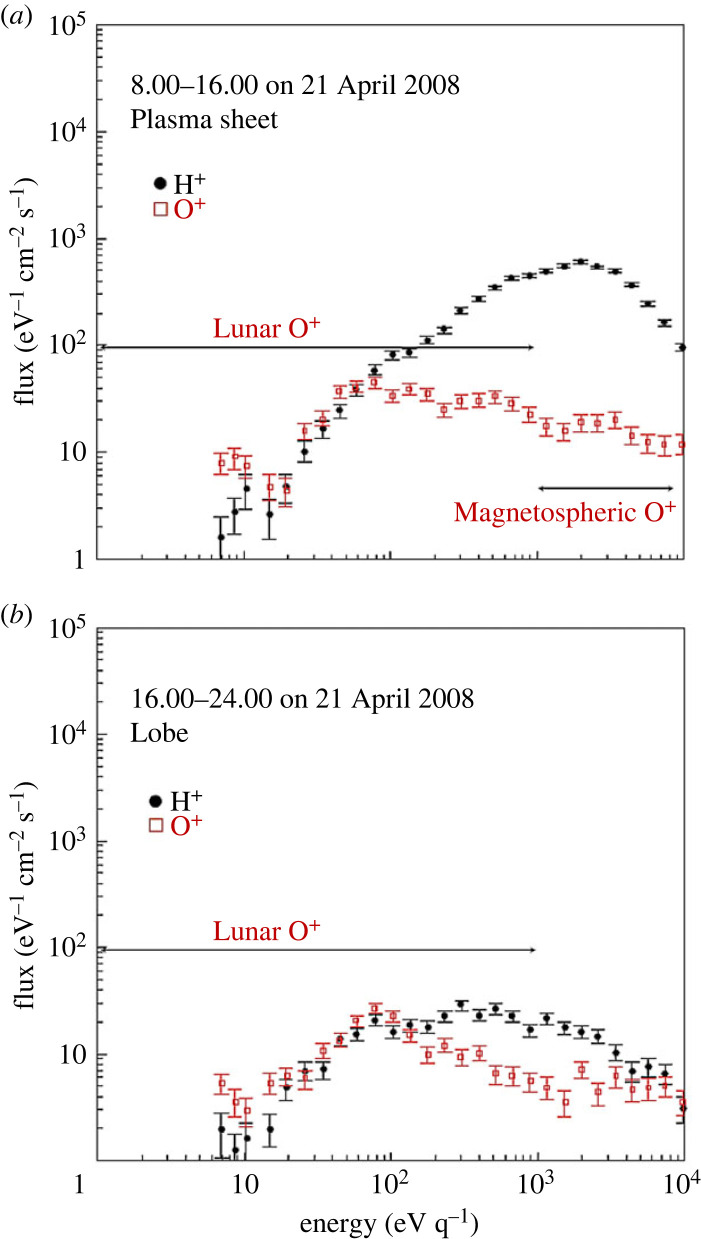
Energy spectra of H^+^ and O^+^ ions measured by the IMA sensor onboard the Kaguya Lunar Orbiter in the terrestrial magnetotail. During the plasma sheet encounter (*a*) there is an enhancement of high-energy (1–10 keV) O^+^ ions, in comparison to those measured in the magnetotail lobe (*b*). The calculated density and net flux of these magnetospheric O^+^ ions, during the plasma sheet encounter, were 1.2 × 10^−3^ cm^−3^ and 2.6 × 10^4^ cm^−2^ s^−1^ respectively. (From: [23]).

O^+^ ion beams of terrestrial origin streaming downtail were observed by the Geotail spacecraft, and at lunar distances they can have fluxes up to approximately 10^4^ ions cm^−2^ s^−1^ [78]. During high geomagnetic activity conditions these beams can include heavy atomic and molecular ions [79,80]. Closer to the Moon, O^+^ downtail streaming beams have been observed by the Kaguya Lunar Orbiter [23]. The spectral characteristics of these downtail streaming O^+^ ions allow a clear separation between the O^+^ ions of lunar origin (few 10 eV to approx. 100 eV) and the terrestrial magnetospheric O^+^ ions (few keV), cf. figure 5 (adapted from [23]). Simulations of the trajectories of these heavy ions show how they can be ejected from the terrestrial ionosphere during high geomagnetic activity conditions and then they propagate downtail, reaching energies of several keV to several 10 keV at lunar distances [18,22].

Bursts of energetic electrons (BEE) constitute another transient population to which the Moon can be exposed if, during high activity periods, it is located in the terrestrial magnetotail or in the magnetosheath [81].

## 3. Energetic particle interaction with the Moon

Energetic particles arriving at the Moon interact with the tenuous lunar exosphere and the lunar regolith.

**Figure 6.**
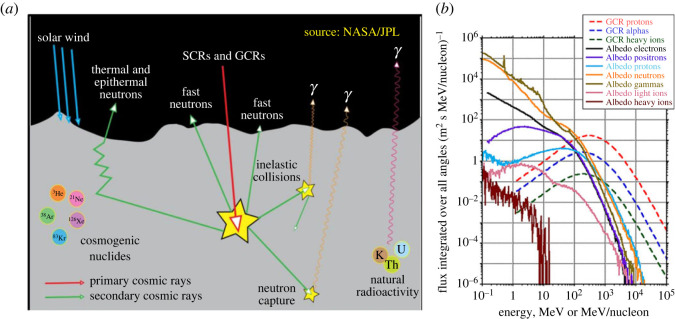
(*a*) Schematic of the solar wind and GCR interaction with the lunar regolith. (Credit: NASA/JPL). (*b*) Energy spectra of pristine GCR species (dashed lines) and of lunar albedo species (continuous lines), calculated with the Geant4 simulation toolkit. (From: [82]).

SEPs and GCRs arriving at the Moon's surface can be absorbed, or scattered, or can remove another particle from the lunar regolith, or can produce cosmogenic nuclides [36,82], cf. figure 6. Their interaction with the lunar regolith produces albedo energetic particles, which with current instruments can be detected and resolved up to a few 100 MeV. Albedo energetic particles include hydrogen nuclei (protons, deuterons, tritium ions), heavier ions, electrons, positrons, neutrons, gamma-ray photons and also muons and pions [83].

Gamma-ray photons can be emitted due to inelastic scatter, neutron capture or natural or induced radioactivity. The emitted gamma-ray lines are characteristic of the elemental composition of the surface and near-subsurface of the regolith (down to a few 10 cm) [84,85].

Neutrons are produced from the interaction of SEPs and GCRs with the regolith material [82,86,87]. Albedo neutron fluxes are particularly sensitive to the abundance of hydrogen atoms in the regolith, and they are used for the identification of hydrogen in the form of water ice [88,89]. The albedo neutrons contribute substantially to the radiation risks for humans, due to the mode the neutrons interact with the body tissues [90].

**Figure 7.**
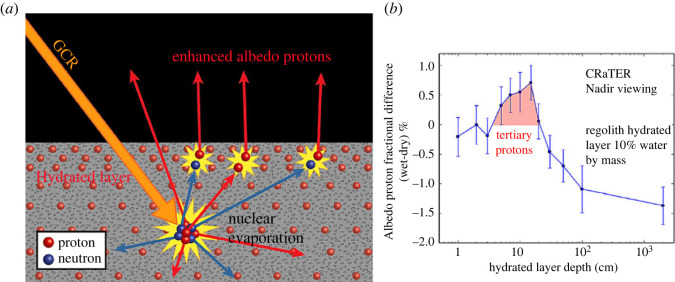
(*a*) Schematic representation of the effects of a hydrated layer of lunar regolith in the production of GCR albedo (secondary) protons. The nuclear evaporation process from deep in the regolith produces abundant secondary particles in all directions. (From: [37]). (*b*) Geant4 simulations of albedo protons from the wet versus dry regolith, due to GCR bombardment. The fractional difference in the proton albedo flux of the wet versus dry regolith is shown as a function of the depth of the hydrated layer. The wet regolith consists of 10% hydrated material by mass. (From: [37]).

The fluxes of the albedo energetic protons are also in their turn sensitive to the regolith hydration [37,82,83], cf. figure 7. The separation of the pristine energetic particle fluxes from the albedo energetic particles (e.g. by zenith centred/nadir centred detector looking directions respectively, cf. §4.b) can thus provide information on the deep space SEP and GCR environment and on the interaction of the lunar regolith with this environment.

SEPs and GCRs interacting with the nuclei of atoms in the lunar regolith can produce cosmogenic nuclides. The concentrations of these cosmogenic nuclides give critical information for dating lunar soil samples [91,92], for providing a record of the long-term evolution of the solar activity [36], or for tracing the history of surface exposure/burial of the different lunar soil grains, resulting from the regolith ‘gardening’ [93].

GCRs are also capable of driving chemical reactions on exposed ice, which can lead to the formation of more complex molecules [94,95]. Permanently shadowed regions (PSRs), which act as cold traps and are thus expected to hold surface ice deposits, are sites of particular interest for such GCR-induced ice chemistry.

Solar wind protons arriving at the Moon's surface can similarly be absorbed, or scattered, or can remove another atom from the lunar regolith by sputtering or desorption [41,96–99]. It results that a large fraction of the solar wind protons, up to 20%, is reflected back to space as neutral hydrogen atoms (ENAs: Energetic Neutral Atoms). The parent solar wind ions undergo an average energy loss of ≥50% when reflected as ENAs [96]. The rougher the surface of the regolith, on which this process occurs, the deeper the penetration of the solar wind ions inside the regolith, which in turn decreases the fraction of reflected particles [100,101].

**Figure 8.**
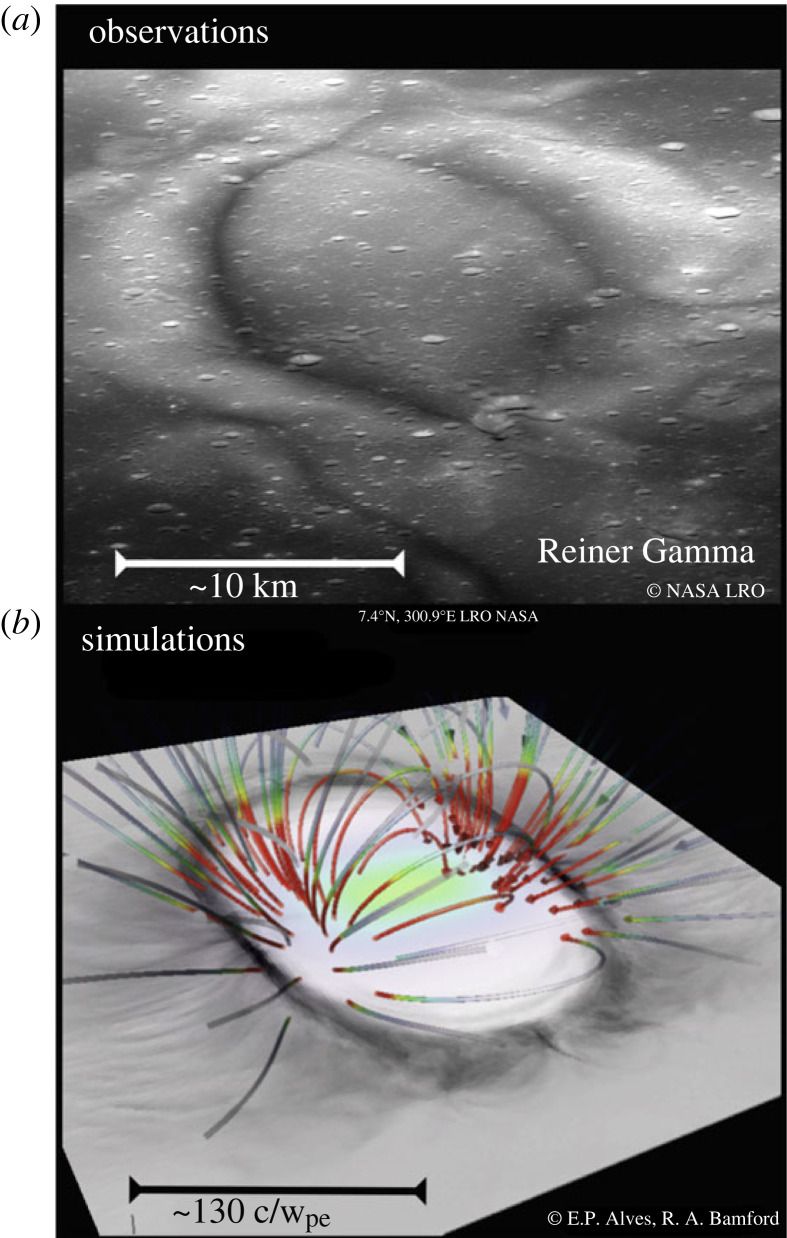
(*a*) Lunar Reconnaissance Orbiter (LRO) image of the central region of the Reiner Gamma Formation lunar swirl. (*b*) A slice of the relative solar wind proton density above this lunar swirl obtained from a three-dimensional simulation, with the initial magnetic field lines corresponding to a single subsurface dipole. (From: [50]).

As the solar wind proton trajectories are modulated by the surface electrostatic potential and by the eventual local magnetic field anomalies (cf. figure 8), detection and imaging of these reflected ENAs provides a way to investigate the lunar surface electric and magnetic fields [42,50,102,103].

Some of the solar wind protons interacting with the Moon's surface could even be scattered and reflected back as negative ions [4]. Such negative ions can be produced by charge inversion processes during the energetic proton scattering and they are weakly bound systems, having a short lifetime due to photo-detachment by the solar radiation [104]. Negative ions have been observed in the ionospheres of Earth, comets, Titan and of the icy moons of Saturn [105–108].

Sputtering is another process induced by the exposure of the lunar regolith to the solar wind ions and SEPs [26]. This process releases different species from the surface into space, contributing to the population of the lunar exosphere, particularly with the heavier more refractory elements [24,109,110]. During CMEs, the exposure of the regolith to the SEPs and to the CME driver gas, which is highly enriched in heavy ions, can result in a more than tenfold increase of the Na, K, Ca and Mg atoms populating the exosphere, compared to their background densities [71].

Local crustal magnetic anomalies (or ‘swirls’) constitute ‘mini-magnetospheres’, shielding locally the lunar regolith from the solar wind protons and from the resulting space weathering [49,50,111–115]. GCRs, however, due to their much higher energies are largely unaffected by these local magnetic anomalies.

Those solar wind protons that do not scatter back, but are absorbed in the lunar regolith (top 20–30 nm of the lunar grains), diffuse within the regolith. There they can interact with the oxygen atoms in the regolith and form OH [35,44,45]. These hydroxyl radicals, produced by the solar wind protons, contribute to the formation and to the release of water molecules, and thus to a water cycle on the Moon driven by the solar wind [4,116–119]. This water formation cycle appears to be a fast process, following the lunar diurnal cycle [120].

In addition to the solar wind implantation being one of the primary mechanisms leading to the formation of water (OH/H_2_O) in the lunar regolith, high fluxes of energetic electrons from the terrestrial plasma sheet (greater than 1 keV) have also been suggested as a mechanism for the production of lunar surface water [121]. In this case, when the Moon crosses the Earth's magnetotail the plasma sheet energetic electrons would produce defects in the regolith grains, in the form of non-bonding oxygen atoms, allowing any trapped H atoms to react with the defects and form hydroxyl radicals or water molecules.

The lunar surface exposure to the sunlight and to the flux of charged particles results also in an electrostatic surface charging [122]. An electric potential develops between the lunar surface and the ambient plasma, which manifests as a near-surface plasma sheath having a scale height of the order of the Debye length [123–125]. In the vicinity of the terminator, this near-surface electric field changes from mostly positive (few 10 V) on the dayside, due to photoelectron emission, to highly negative (of the order of the ambient electron temperature, i.e. up to several −100 V) on the nightside [126]. The topographical features are another element driving the local electrostatic potential, as for example is the case close to shadowed craters or at the walls of deep cavities [64,127,128]. Surface charging is also highly sensitive to the energetic electron environment. During an event of exposure to bursts of energetic electrons (0.1–2.0 MeV), a large lunar surface charging of ∼−5.4 kV was observed by Chang'E-1 [81].

Energetic charged particles, as SEPs and GCRs, can even penetrate deeper within the lunar regolith and produce deep dielectric charging [129]. This charging depends not only on the SEP and GCR energy and fluxes, but also on the regolith's electrical conductivity and permittivity. Intense SEP events produce transient but strong subsurface electric fields (≥10^6^ V m^−1^), that could induce dielectric breakdown, mostly within permanently shadowed regions (PSRs). These breakdown events rapidly vaporize small, filamentary conduits within the dielectric, possibly resulting in significant modifications of the physical and chemical properties of the lunar regolith. Such ‘breakdown weathering’ could even increase the portion of fine monomineralic grains within the PSRs [130], consistently with the increased porosity observed within these permanently shadowed regions [131].

Dust constitutes another element of the lunar plasma environment [54,132,133]. Dust grains can either be ejected from the regolith following the impact of interplanetary micrometeoroids, or can be electrostatically levitated by the grain charging, as discussed above. This results in a dusty plasma system containing neutral atoms and molecules of the lunar exosphere, solar-wind ions and electrons, ions and electrons from the Earth's magnetosphere (when the Moon gets within the terrestrial magnetotail), photoelectrons from the interaction of the solar radiation with the lunar surface, and charged dust grains soaring above the lunar surface.

## 4. The Lunar Orbital Platform - Gateway

The Lunar Orbital Platform - Gateway (or simply Gateway) is a versatile space station that will be assembled on the Moon's orbit by NASA and its international partner organizations, including ESA, JAXA, and CSA. It will provide support for lunar activities, including the Artemis program to return humans to the Moon [67,134]. The Gateway will also offer new opportunities for fundamental and applied scientific research in a variety of disciplines [135].

The initial components of the Gateway (Phase 1) will be launched into a halo orbit around the Moon (3200 × 70 000 km, 90° inclination) no earlier than 2025. Additional modules will be launched during the late 2020s (Phase 2).

### (a) Space plasmas and energetic particles measurement opportunities from the Lunar Orbital Platform - Gateway

During Phase 1 a set of radiation/space weather and heliophysics instruments will be flown on the Gateway: the ‘Heliophysics Environmental and Radiation Measurement Experiment Suite’ (HERMES) from NASA, the ‘European Radiation Sensors Array’ (ERSA) from ESA, and the ‘Internal Dosimeter Array’ (IDA) from ESA and JAXA [67,136].

In preparation of the Gateway Phase 2 scientific payload, ESA established international science teams to assist in the definition of payload studies, including a topical team in the field of space plasma physics. The ‘Space Plasma Physics Science opportunities for the Lunar Orbital Platform-Gateway’ topical team was set up by ESA in 2019 and identified the science objectives that can be addressed from onboard the Gateway, in the field of space plasma physics and energetic particles. It then defined the physical parameters needed to be measured in order to address these objectives, and the instrumentation necessary to perform these measurements [137].

Table 1 provides a synthesis of the science questions identified by the topical team, that can be addressed from instrumentation onboard the Gateway, and shows how each of them converts into a measurement requirement, and then to the corresponding instrument/payload requirement.

**Table 1.** Science objectives that can be addressed from onboard the Gateway, in the field of space plasma physics and energetic particles, and corresponding measurement and instrumentation requirements. (From: [137]).

science objective	measurement requirement	*in-situ* measurements instrument	remote sensing instrument
monitor solar wind as a driver for the dynamics of terrestrial magnetosphere, terrestrial and lunar exospheres, lunar surface sputtering and charging	solar wind density and transport velocity	Faraday Cup	—
		1–10^2^ cm^−3^, 0.1–40 keV ions	electrostatic analyser	
		200–1000 km s^−1^, Δ*E/E* < 17%		
		IMF: 100 nT instrument range 0.1 nT resolution	magnetometer	—
monitor and characterize	40 keV – 100 MeV ions (SEPs)	energetic particle detectors	MeV ENA imager
SEPs and GCRs for radiation environment and as lunar surface sputtering sources	up to approximately 5 GeV (GCRs)		
	50 MeV/nucleon for composition		
	approximately 40 keV – approximately 30 MeV electrons		
monitor and characterize the response of the terrestrial magnetosphere to the solar wind with a wide coverage of geospace	detect and image solar wind charge exchange X-rays	—	soft X-ray imager
	0.2–2.0 keV, FOV 10° × 10°		
	ang. resol.: 0.3 R_E_ from Moon		
	detect and image terrestrial magnetosphere ENAs		ENA imager
	approximately 1–300 keV, FOV approximately 20° × 20°		
monitor solar wind interaction with the lunar exosphere, regolith and magnetic anomalies	detect and image low-energy ENAs: 0.1–10 keV,	—	LENA imager
	30% *ΔE*/*E*, FOV ∼ 20° × 20°,		
	approximately 5° resolution		
reveal the solar wind ion dynamics in the vicinity of the lunar magnetic anomalies	detect and image low-energy ENAs:	—	LENA imager
	0.01–3 keV,		
	30% *ΔE*/*E*, FOV ∼ 5° × 120°,		
	approximately 5° resolution		
monitor the terrestrial and lunar exospheres, plasmasphere	detect and image	ion mass spectrometer (lunar pickup ions)	UV/EUV spectro-imager
	EUV emissions		
	30.4, 83.6, 121.6 and 130.4 nm		
	approximately 5 arcmin resolution		
monitor ambient plasma in different environments (solar wind/magnetosheath/terrestrial magnetotail/lunar wake)	plasma density and temperature	Langmuir probe Ion mass spectrometer electron spectrometer	—
	approximately 0.01–40 keV, 10^−3^–10^2^ cm^−3^		
	ion composition: *m*/*Δm* > 15		
	magnetic field: 1000 nT range	magnetometer	—
monitor magnetospheric and planetary radio emissions	AC electric and magnetic field		radio instrument

### (b) Conceptual space plasmas and energetic particles instrument package

Following the determination of the science objectives and of the measurement requirements by the ESA topical team, and then of an invitation to tender issued by ESA, a conceptual design study was undertaken for a ‘Space Plasma Physics Payload Package onboard the Gateway’ (SP4GATEWAY), addressing these objectives while being compatible with the requirements. This is a notional study, and it included a suite of 13 instruments, for both *in-situ* and remote sensing measurements, particles and fields [137]. The particle detection instruments have complementary energy ranges, providing an almost continuous energy coverage from approximately 10 eV up to the approximately GeV energy range. This conceptual design study provided also the suggested accommodation of the instruments on the Gateway, taking into account the various constraints: pointing requirements for instruments with a field-of-view (FOV), unobstructed fields-of-view, instrument placing on areas with low electrostatic charging, instrument grouping when possible. Here we present the main results, while focusing on the energetic particle instrumentation (cf. also table 1).

**Figure 9.**
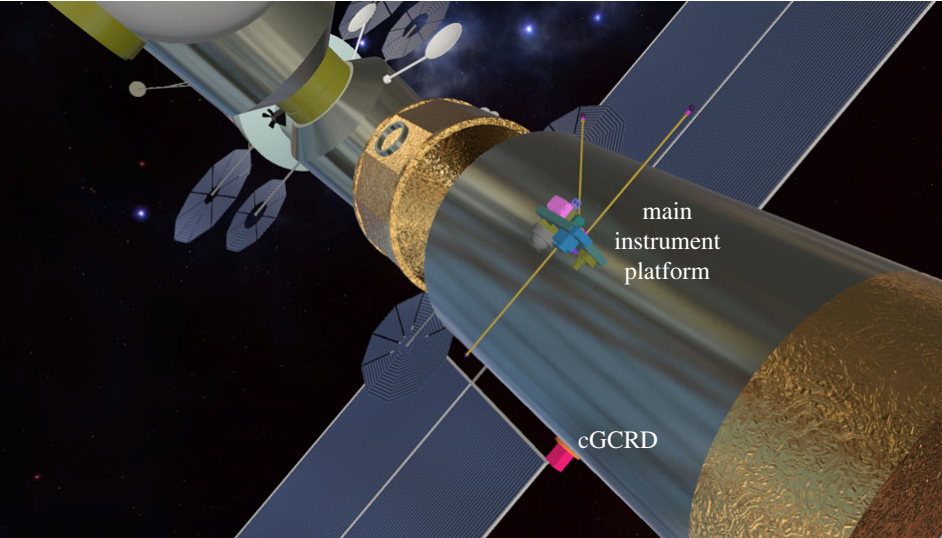
The two-sided Main Instrument Platform mounted on the Logistics Module of the Gateway, accommodating most of the *in-situ* measurement space plasma instruments. The ‘magenta cube’, on the side of the Logistics Module, is the standalone GCR instrument (cGCRD). (From: [137]).

Most of the *in-situ*-measurement instruments have been grouped on a platform, mounted on the Logistics Module of the Gateway and directly exposed to the solar wind flux (figure 9). Of particular interest for energetic particle measurements are:

— Two solar wind instruments: an electrostatic analyser solar wind ion energy spectrometer and a solar wind Faraday cup. They will provide the solar wind proton velocity distribution functions and the solar wind density, velocity, temperature and alpha particle content.
— A magnetospheric ion mass spectrometer, using a combination of electrostatic analyser and of a time-of-flight mass analysis, based on a grazing-incidence MCP [138]. It will cover the approximately 10 eV e^−1^–approximately 40 keV e^−1^ energy range and will provide the composition and velocity distribution functions of the ambient plasma ions: ions of terrestrial magnetosphere origin, pickup ions from the lunar exosphere and solar wind ions.
— A magnetospheric electron spectrometer (approx. 5 eV–approx. 20 keV energy range), for measuring the velocity distribution functions of the solar wind electrons (pristine or reflected from lunar crustal magnetic field anomalies), and of the terrestrial magnetosphere plasma sheet electrons.
— An energetic particle detector, to detect and measure the fluxes of the energetic particles, ions and electrons: SEPs, low-energy GCRs and terrestrial plasma sheet energetic particles. The instrument will also help to analyse the spectra of the secondary high-energy ions, released from the lunar regolith following its exposure to GCRs and/or SEPs (albedo energetic particles, cf. §3). It will cover the approximately 40 keV – approximately 100 MeV energy range for ions and approximately 20 keV–approximately 30 MeV for electrons and provide a Δ*E* ≤ 10 keV energy resolution. This instrument will also supply, for ions, a measure of their composition (protons to iron nuclei). In order to cover both pristine and albedo energetic particles, it will have two identical detection heads, each with a 60° × 60° FOV: one pointing to the lunar zenith, to detect pristine energetic particles, and the other pointing to the opposite direction, i.e. the lunar nadir, for detecting the albedo particles.
— A Galactic Cosmic Ray detector, to provide the spectra and the composition of the GCRs and the SEPs, covering the 0.1 to approximately 5 GeV energy range and with a Δ*E*/*E* ≤ 30% energy resolution. This instrument will be complementary to the energetic particle detector, covering the higher energies. The proposed instrument is the Mini.PAN penetrating particle analyser, derived from the Penetrating particle ANalyzer (PAN) developed for deep space applications [139]. Mini.PAN is based on the particle detection principle of a magnetic spectrometer, with novel layout and detection concepts to optimize the measurement precision for both high flux and low flux particles. In Mini.PAN the deflection of the particle in the magnetic field is measured by precise silicon strip tracking detectors, while the elemental composition of the particle is determined by its charge and *Z*, which is measured with the d*E*/d*x* method at multiple points. Mini.PAN is designed to precisely measure the momentum, charge and the direction of energetic particles between 100 MeV nuc^−1^ and a few GeV nuc^−1^.

The orientation of the fields of view (FOVs) of these instruments, required to satisfy the observational requirements, was then examined by simulating how the FOV of each sensor head evolves along the Gateway orbit.

**Figure 10.**
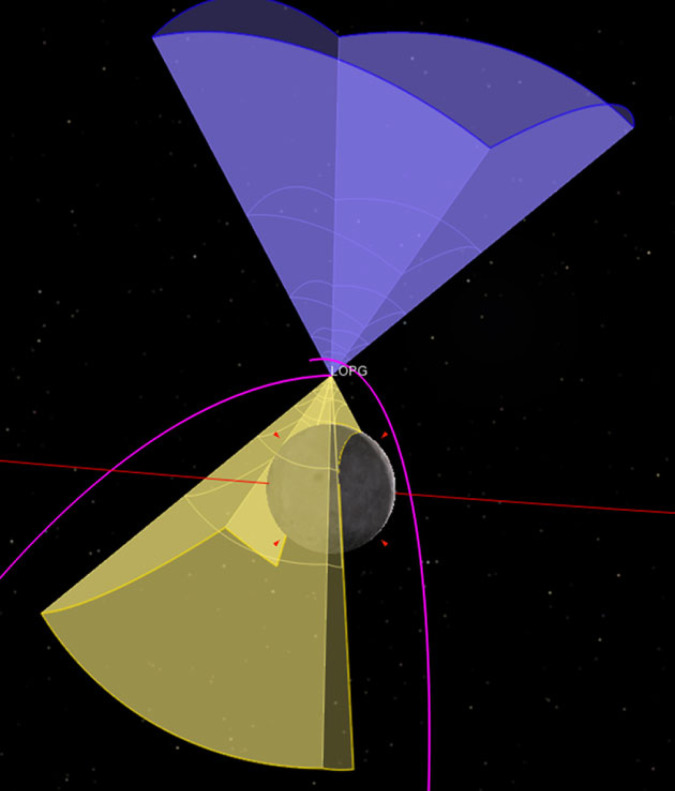
Instantaneous FOVs of the two oppositely directed sensor heads of the energetic particle detector instrument, near periapsis. Purple (upward pointing) FOV: pristine energetic particle flux. Yellow (downward pointing) FOV: Moon albedo energetic particle flux. The magenta line is the track of the centre of the FOV along the Gateway orbit. (From: [137]).

The FOVs of the two oppositely directed sensor heads of the energetic particle detector instrument, near a periapsis pass, are presented in figure 10. As shown there, one of the two oppositely-directed sensor heads is oriented towards the local zenith, and gets an unobstructed view to the pristine energetic particle flux (purple FOV), whereas the other sensor head is oriented towards the local nadir and the albedo energetic particles from the Moon dominate its FOV (yellow FOV). In this way the instrument separately measures both populations, pristine and albedo high-energy particles.

**Figure 11.**
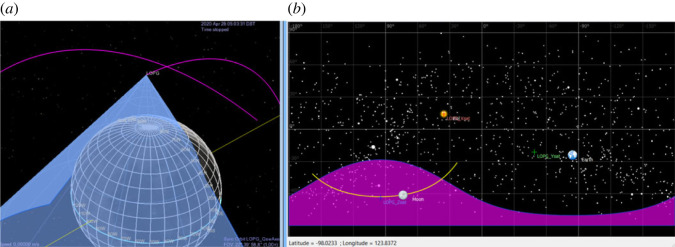
FOV of the Galactic Cosmic Ray detector. (*a*) FOV near periapsis (light blue cone), dominated then by the albedo GCR particles from the Moon (grid sphere). (*b*) Projection on the sky of the FOV along the Gateway orbit (magenta area). (From: [137]).

For the Galactic Cosmic Ray detector, which is a single sensor head instrument, the FOV near a periapsis pass is shown in figure 11, left panel (light blue FOV). It appears that, near periapsis, the FOV covers mainly the albedo GCR particles from the Moon. However, during most of the remaining orbit (right panel) this instrument gets an unobstructed view to the open sky and provides access to the pristine GCR environment.

## 5. Conclusion

The Moon and its space environment constitute an ideal system to study deep space plasma, energetic particles and their interaction with an airless and unmagnetized planetary body. The solar wind, solar energetic particles, galactic cosmic rays and the terrestrial magnetotail plasma interact with the lunar regolith and the tenuous lunar exosphere constituting a complex, multi-scale interacting system. The study of this system allows a series of scientific objectives to be addressed, several of which have a multi-disciplinary dimension:

— Planetary and terrestrial space weather, through the analysis of the effects of the solar activity on the lunar space plasma environment and on the terrestrial magnetotail plasma.
— Radiation physics, through the analysis of the energy and mass spectra of the high-energy particles in the lunar environment, particularly in view of the upcoming Artemis missions and the related radiation risks for the astronauts.
— Studying the composition and the hydration level of the lunar regolith through the analysis of the fluxes of the albedo energetic particles, which result from the interaction of the precipitating energetic particles with the regolith.
— Studying the solar wind-induced water cycle on the Moon, through the implantation of solar wind protons into the lunar regolith and their interaction with the oxygen atoms, producing hydroxyl radicals that contribute to the formation and to the release of water molecules.
— Studying the implantation of solar energetic particles and galactic cosmic rays into the lunar regolith, and the production of cosmogenic nuclides.
— Understanding the mini-magnetospheres that form above the local magnetic anomalies (or ‘swirls’) of the Moon, and which are probably the smallest natural magnetospheres in the Solar System.
— Understanding the complex electric fields and electrostatic dust levitation mechanisms, which are the manifestation of the interaction of the Moon surface with its space plasma environment.
— Studying the escape of heavy ions from the terrestrial ionosphere, during high geomagnetic activity conditions, their implantation into the lunar regolith and their role as ‘recorders’ of the long-term evolution of the terrestrial atmosphere (and of its habitability).

## Data Availability

This article has no additional data.
